# Identification of Key mRNAs and lncRNAs in Neonatal Sepsis by Gene Expression Profiling

**DOI:** 10.1155/2020/8741739

**Published:** 2020-08-25

**Authors:** Lin Bu, Zi-wen Wang, Shu-qun Hu, Wen-jing Zhao, Xiao-juan Geng, Ting Zhou, Luo Zhuo, Xiao-bing Chen, Yan Sun, Yan-li Wang, Xiao-min Li

**Affiliations:** ^1^Department of Emergency Medicine, First People's Hospital of Lianyungang, Hospital of the Clinical Medical School of Nanjing Medical University, Lianyungang 222002, China; ^2^Department of Intensive Care Unit, Affiliated Hospital of Xuzhou Medical University, Xuzhou 221000, China

## Abstract

Neonatal sepsis is one of the most prevalent causes of death of the neonates. However, the mechanisms underlying neonatal sepsis remained unclear. The present study identified a total of 1128 upregulated mRNAs and 1008 downregulated mRNAs, 28 upregulated lncRNAs, and 61 downregulated lncRNAs in neonatal sepsis. Then, we constructed PPI networks to identify key regulators in neonatal sepsis, including ITGAM, ITGAX, TLR4, ITGB2, SRC, ELANE, RPLP0, RPS28, RPL26, and RPL27. lncRNA coexpression analysis showed HS.294603, LOC391811, C12ORF47, LOC729021, HS.546375, HNRPA1L-2, LOC158345, and HS.495041 played important roles in the progression of neonatal sepsis. Bioinformatics analysis showed DEGs were involved in the regulation cellular extravasation, acute inflammatory response, macrophage activation of NF-kappa B signaling pathway, TNF signaling pathway, HIF-1 signaling pathway, Toll-like receptor signaling pathway, and ribosome, RNA transport, and spliceosome. lncRNAs were involved in regulating ribosome, T cell receptor signaling pathway, RNA degradation, insulin resistance, ribosome biogenesis in eukaryotes, and hematopoietic cell lineage. We thought this study provided useful information for identifying novel therapeutic markers for neonatal sepsis.

## 1. Introduction

Neonatal sepsis was a severe systematic infectious disease in neonates induced by bacteria, fungi, and viruses [1]. Neonatal sepsis is one of the most prevalent causes of death of neonates [2]. Adult sepsis has been studied in depth, but many abundant studies stated that the neonatal immune response to sepsis is different from adults; comparable research on neonatal vascular endothelium is not enough. Neonatal endothelial cells expressing lower amounts of adhesion molecules show a reduced capacity to reactive oxygen species [3]. In the past decades, emerging studies showed activation of lymphocytes, neutrophils, and mononuclear macrophages played crucial roles in the progression of neonatal sepsis. A few genes were identified to be associated with neonatal sepsis. For example, TLR2 and TLR4 were associated with the recognition of the bacteria in neonates [4]. PIK3CA, TGFBR2, CDKN1B, KRAS, E2F3, TRAF6, and CHUK were reported to be key regulators in neonatal sepsis [5]. However, the detailed mechanisms underlying these processes remained elusive.

Long noncoding RNAs (lncRNAs) were a class of ncRNAs longer than 200 bps. Emerging studies showed lncRNAs were important regulators in multiple human diseases such as diabetes, cancers, and neonatal sepsis. lncRNAs regulate target expression in different levels, including transcriptional and posttranscriptional levels. lncRNAs could bind to RNA, protein, and DNA molecules in cells. Very few reports are aimed at elucidating the functions and roles of lncRNAs in neonatal sepsis. Until now, only one report showed lncRNA SNHG16 reverses the effects of miR-15a/16 on the LPS-induced inflammatory pathway in neonatal sepsis [6]. Exploring the roles of lncRNAs in neonatal sepsis could provide novel clues for us to understand the mechanisms underlying this disease progression.

The previous study is aimed at identifying differently expressed mRNAs and lncRNAs in neonatal sepsis by analyzing GSE25504 [7]. Protein-protein interaction network and coexpression network analysis were used to identify key mRNAs and lncRNAs. Bioinformatics analysis was also conducted to predict the potential roles of these genes in neonatal sepsis. This study could provide novel clues to understand the mechanisms of underlying neonatal sepsis progression.

## 2. Materials and Methods

### 2.1. Microarray Data

Three gene expression profile GSE25504 [8] was downloaded from the GEO database. GSE25504, which was based on the GPL6947 platform, was submitted by Dickinson et al. The GSE25504 dataset contained 38 negative blood culture result samples and 25 positive blood culture result samples. The analysis for differential gene expression between tumor and normal tissue was performed using GeneSpring software version 11.5 (Agilent Technologies, Inc., Santa Clara, CA, USA). Student's *t*-test was used to identify DEGs with an alteration of ≥2-fold. *p* < 0.05 was considered to indicate a statistically significant difference. We applied Limma package to identify DEGs with R software [9].

### 2.2. Coexpression Network Construction and Analysis

In this study, Pearson's correlation coefficient of differently expressed gene- (DEG-) lncRNA pairs was calculated according to the expression value of them. The coexpressed DEG-lncRNA pairs with the absolute value of Pearson's correlation coefficient ≥ 0.8 were selected, and the coexpression network was established by using Cytoscape software.

### 2.3. Pathway Enrichment Analysis

Pathway analysis was used to find the significant pathways according to Kyoto Encyclopedia of Genes and Genomes (KEGG). Fisher's exact test was adopted to select the significant pathways, and the threshold of significance was defined by FDR and *p* value. Significant pathways were extracted according to the thresholds of *p* < 0.05 and intersection gene count > 1.

### 2.4. Integration of the Protein-Protein Interaction (PPI) Network

The Search Tool for the Retrieval of Interacting Genes version 10.0 (STRING: http://string-db.org) [10] was used for the exploration of potential DEG interactions at the protein level. The PPI networks of DEGs by STRING were derived from validated experiments. A PPI score of >0.4 was considered significant. The PPI networks were visualized using Cytoscape software [11] (http://www.cytoscape.org). *p* < 0.05 was considered to indicate a statistically significant difference.

## 3. Results

### 3.1. Identification of Differently Expressed mRNAs and lncRNAs in Neonatal Sepsis by Analyzing Whole Blood Expression Profiling

**Figure 1:**
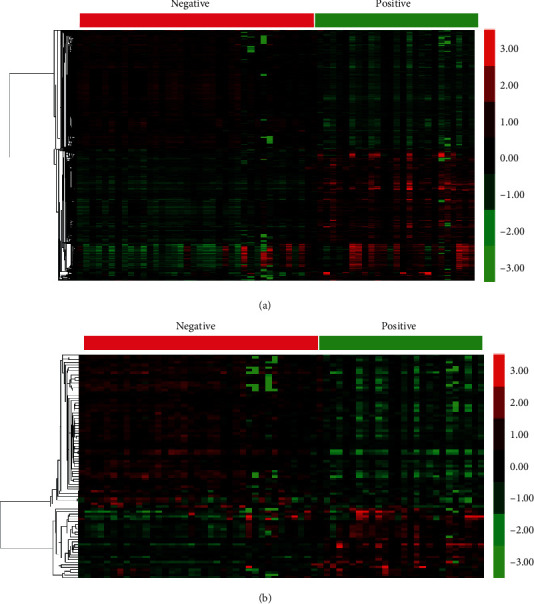
Identification of differently expressed mRNAs and lncRNAs in neonatal sepsis by analyzing whole blood mRNA expression profiling. (a) Hierarchical clustering analysis showed differential mRNAs expression in negative blood culture result samples and positive blood culture result samples. (b) Hierarchical clustering analysis showed differential lncRNA expression in negative blood culture result samples and positive blood culture result samples.

The present study is aimed at identifying differently expressed mRNAs and lncRNAs in neonatal sepsis by analyzing whole blood mRNA expression profiling, GSE25504, from the NCBI GEO dataset (https://www.ncbi.nlm.nih.gov/geo/query/acc.cgi?acc=GSE25504). A total of 38 negative blood culture result samples and 25 positive blood culture result samples were included in this dataset. As shown in Figures 1(a) and 1(b), 1128 upregulated mRNAs and 1008 downregulated mRNAs with log2 fold change (FC) | ≥1.0 and false discovery rate (FDR) ≤ 0.01 were identified as differently expressed genes (DEGs). Meanwhile, this study identified 28 upregulated lncRNAs and 61 downregulated lncRNAs in positive samples compared to negative samples as differently expressed lncRNAs (DElncs).

### 3.2. PPI Network Analysis of DEGs in Neonatal Sepsis

**Figure 2:**
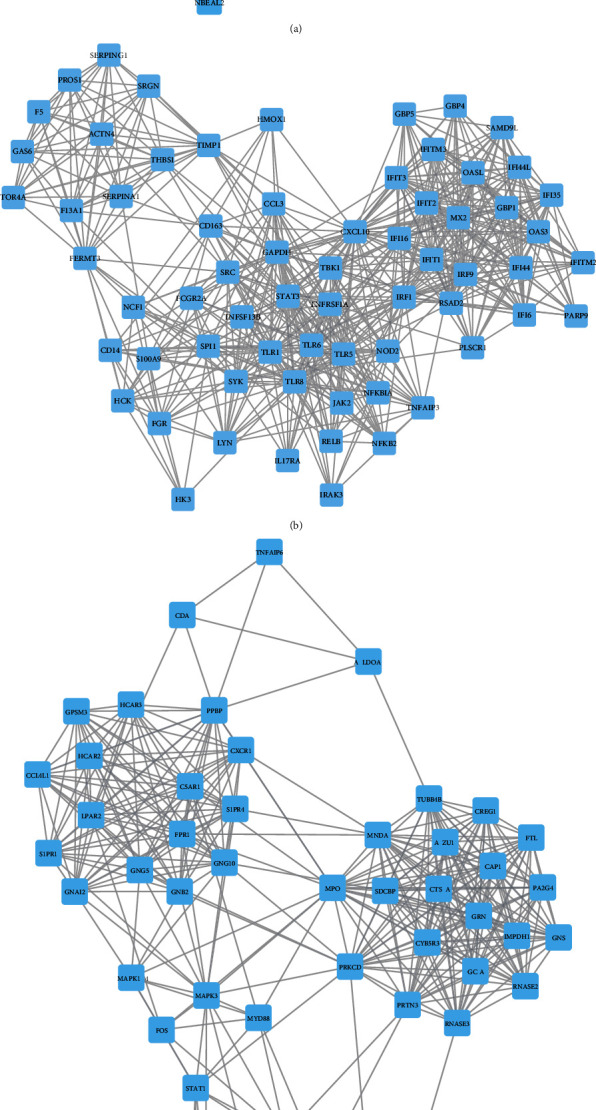
Construction of upregulated PPI networks in neonatal sepsis. PPI network analysis showed upregulated hub PPI network 1 (a), hub PPI network 1 (b), and hub PPI network 3 (c).

**Figure 3:**
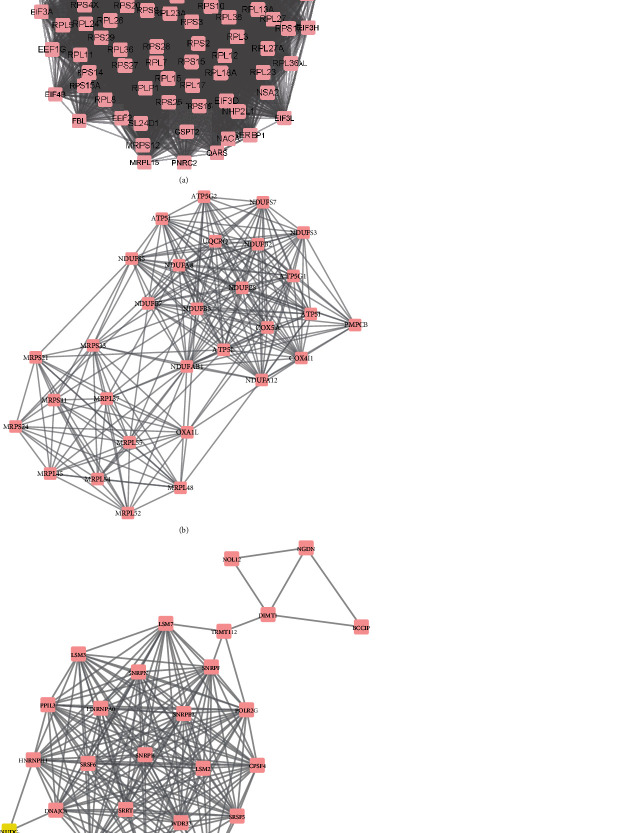
Construction of downregulated PPI networks in neonatal sepsis. PPI network analysis showed downregulated hub PPI network 1 (a), hub PPI network 1 (b), and hub PPI network 3 (c).

The above analysis revealed multiple differently expressed genes in neonatal sepsis. However, the interactions of these DEGs remained largely unclear. To obtain the interactions among the 22 upregulated mRNAs and 863 downregulated mRNAs in the neonatal sepsis, the present study constructed and presented PPI networks using the STRING database and Cytoscape software. The combined score > 0.4 was used as the cut-off criterion. Following the construction of PPI network, a MCODE plug-in analysis was performed to identify hub networks (degree cut-off ≥ 2 and the nodes with edges ≥ 2-core) in the PPI network using Cytoscape software (Figure 2). As shown in Figure 2, upregulated hub network 1 included 71 nodes and 1187 edges, upregulated hub network 2 included 66 nodes and 611 edges, and upregulated hub network 3 included 62 nodes and 529 edges. As shown in Figure 3, downregulated hub network 1 included 94 nodes and 4048 edges, downregulated hub network 2 included 30 nodes and 247 edges, and downregulated hub network 3 included 26 nodes and 199 edges. Blue nodes indicate upregulated genes, and pink nodes indicate downregulated genes in the neonatal sepsis.

Also, we identified several key regulators in these PPI networks. The key regulators in upregulated PPI networks included ITGAM (degree = 131), ITGAX (degree = 101), TLR4 (degree = 100), ITGB2 (degree = 92), SRC (degree = 87), and ELANE (degree = 81). The key regulators in downregulated PPI networks included RPLP0 (degree = 128), RPS28 (degree = 128), RPL26 (degree = 124), RPL27 (degree = 123), NSA2 (degree = 122), RPS15 (degree = 120), RPS10 (degree = 117), RPS13 (degree = 117), RPS20 (degree = 117), RPL36 (degree = 110), FAU (degree = 108), NHP2L1 (degree = 106), RPL23 (degree = 106), RPS25 (degree = 105), RPL9 (degree = 101), RPL30 (degree = 100), and RPL35A (degree = 100).

### 3.3. Bioinformatics Analysis of DEGs in Neonatal Sepsis

**Figure 4:**
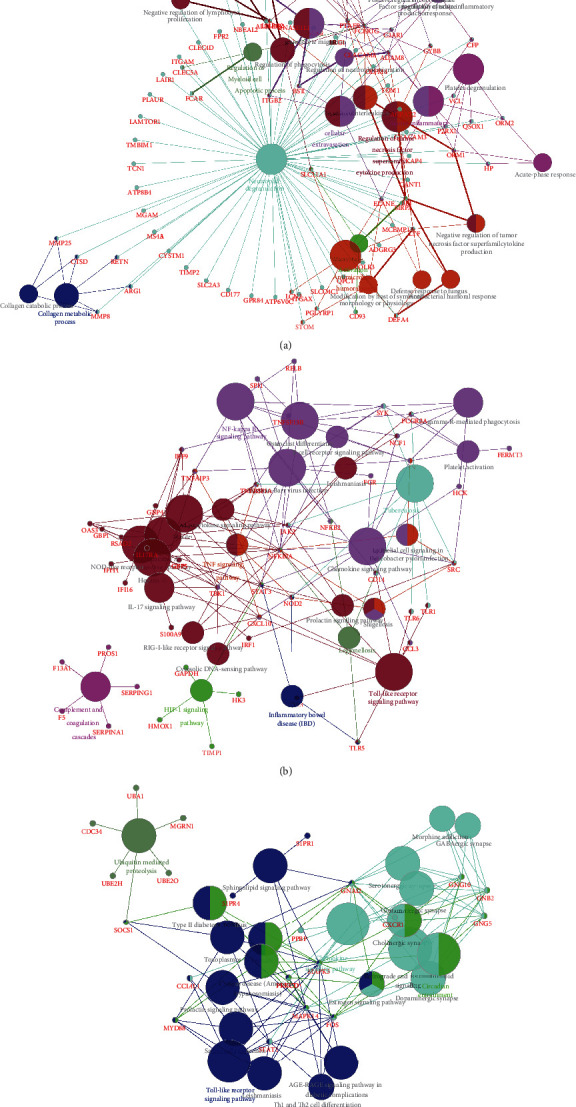
Bioinformatics analysis of hub upregulated PPI networks in neonatal sepsis. Bioinformatics analysis of up-regulated hub PPI network 1 (a), hub PPI network 1 (b), and hub PPI network 3 (c).

Furthermore, we explored the potential functions of DEGSs in neonatal sepsis. We next performed bioinformatics analysis of upregulated and downregulated hub PPI networks in thyroid cancer using Cytoscape's ClueGo plug-in. Only significant biological processes and pathways (*p* ≤ 0.05) were shown. Our results (Figure 4) showed upregulated hub network 1 was involved in regulation of myeloid cell apoptotic process, cellular extravasation, acute inflammatory response, neutrophil degranulation, macrophage activation, antimicrobial humoral response, and collagen metabolic process. Upregulated hub network 2 was involved in regulating NF-kappa B signaling pathway, TNF signaling pathway, HIF-1 signaling pathway, Toll-like receptor signaling pathway, tuberculosis, legionellosis, and complement and coagulation cascades. Upregulated hub network 3 was involved in regulating ubiquitin-mediated proteolysis, Toll-like receptor signaling pathway, chemokine signaling pathway, and circadian entrainment.

**Figure 5:**
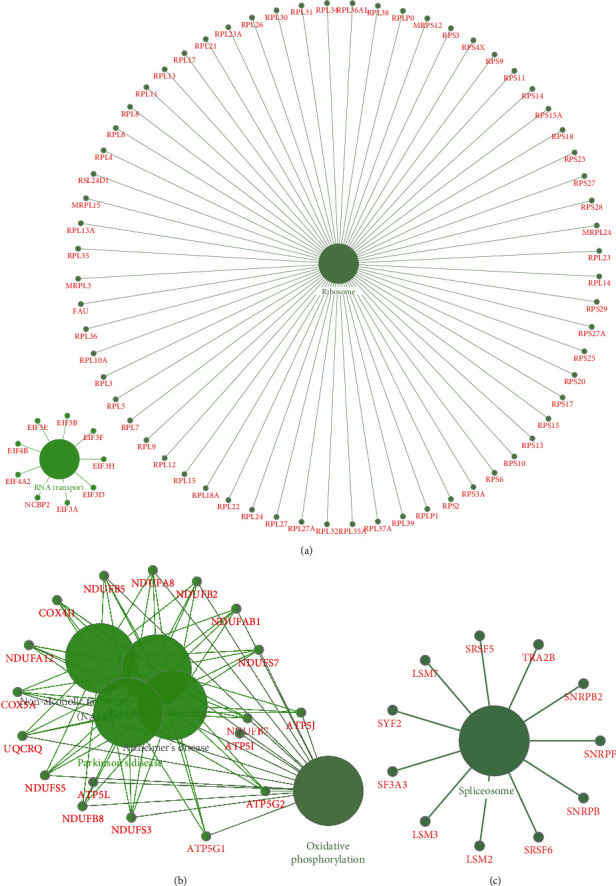
Bioinformatics analysis of hub downregulated PPI networks in neonatal sepsis. Bioinformatics analysis of down-regulated hub PPI network 1 (a), hub PPI network 1 (b), and hub PPI network 3 (c).

Meanwhile, our results (Figure 5) showed downregulated hub network 1 was involved in regulating ribosome, and RNA transport. Downregulated hub network 2 was involved in regulating Parkinson's disease and oxidative phosphorylation. Downregulated hub network 3 was involved in regulating spliceosome.

### 3.4. Coexpression Network Analysis of DElncs in Neonatal Sepsis

**Figure 6:**
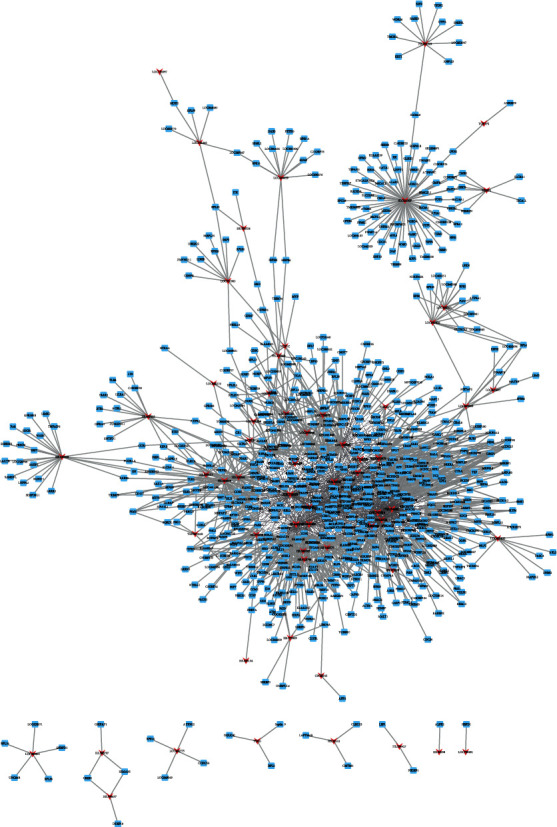
Construction of differently expressed lncRNAs regulating coexpression networks in neonatal sepsis.

We next explored the interactions between mRNAs and lncRNAs. We performed Pearson's correlation calculation of lncRNA-mRNA pair in neonatal sepsis. Based on the correlation analysis results, we constructed an mRNA-lncRNA coexpression network, including 62 lncRNAs, 726 mRNAs, and 2041 interactions between lncRNAs and mRNAs (*p* value < 0.05 and absolute value of correlation coefficient > 0.85). Eight lncRNAs are significantly associated with more than 100 genes, suggesting their key roles in this network (Figure 6), including HS.294603 (degree = 195), LOC391811 (degree = 179), C12ORF47 (degree = 168), LOC729021 (degree = 155), HS.546375 (degree = 151), HNRPA1L-2 (degree = 127), LOC158345 (degree = 100), and HS.495041 (degree = 100).

### 3.5. Bioinformatics Analysis of DElncs in Neonatal Sepsis

**Figure 7:**
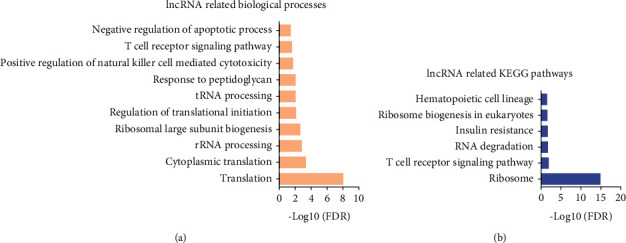
Bioinformatics analysis of differently expressed lncRNAs regulating coexpression networks in neonatal sepsis. (a, b) GO and KEGG analysis of differently expressed lncRNAs in neonatal sepsis.

Bioinformatics analysis for DElncs in neonatal sepsis was also conducted. GO analysis (Figure 7) showed that differentially expressed lncRNAs were associated with translation, cytoplasmic translation, rRNA processing, ribosomal large subunit biogenesis, regulation of translational initiation, tRNA processing, response to peptidoglycan, positive regulation of natural killer cell mediated cytotoxicity, T cell receptor signaling pathway, and negative regulation of apoptotic process. KEGG pathway analysis indicated these lncRNAs were associated with ribosome, T cell receptor signaling pathway, RNA degradation, insulin resistance, ribosome biogenesis in eukaryotes, and hematopoietic cell lineage.

## 4. Discussion

Neonatal sepsis is the most common cause of death of new born children with few certainly reported biomarkers. Infections remained to be the main risk factor that causes the neonatal death. In the past decades, only few reports indicated the potential mechanisms underlying the progression of neonatal sepsis. For example, Medzhitov et al. reported that TLR2 and TLR4 were associated with the recognition of bacteria in neonates [12]. Meng et al. identified core regulators involved in the regulation of neonatal sepsis using bioinformatics analysis [13]. Wynn et al. used gene microarray to identify whole genome gene expression change in very low birth weight with neonatal sepsis [14]. The present study is aimed at identifying differently expressed mRNAs and lncRNAs in neonatal sepsis by analyzing GSE25504. A total of 1128 upregulated mRNAs, 1008 downregulated mRNAs, 28 upregulated lncRNAs, and 61 downregulated lncRNAs were identified. Of note, several DEGs identified by this study had also been reported to be associated with neonatal sepsis. For example, IL1R2 and SOCS3 were reported to drive the neonatal innate immune response to sepsis [15]. In order to elucidate the interactions among these DEGs, we constructed upregulated and downregulated genes regulating PPI networks in neonatal sepsis.

Several key genes were identified in neonatal sepsis, including ITGAM, ITGAX, TLR4, ITGB2, SRC, ELANE, RPLP0, RPS28, RPL26, and RPL27. ITGAM (CD11b) was reported as an early diagnostic marker of neonatal sepsis. TLR4 had been reported to be a key regulator in neonatal sepsis [16]. TLR4 was associated with the recognition of the bacteria in neonates [17]. The single-nucleotide polymorphisms (SNPs) in TLR4 were regarded as genetic modulators of infection in neonatal sepsis [18]. Bioinformatics analysis revealed these DEGs were significantly associated with multiple biological processes, including myeloid cell apoptotic process, cellular extravasation, acute inflammatory response, neutrophil degranulation, macrophage activation, antimicrobial humoral response, collagen metabolic process, NF-kappa B signaling pathway, TNF signaling pathway, HIF-1 signaling pathway, Toll-like receptor signaling pathway, tuberculosis, legionellosis, complement and coagulation cascades, chemokine signaling pathway, circadian entrainment, ribosome, RNA transport, and spliceosome. This signaling had been demonstrated to play crucial roles in neonatal sepsis. For example, altered neonatal Toll-like receptor (TLR) function is hypothesized to contribute to the heightened susceptibility to infection and perpetuated inflammation in term and preterm neonates, clinically evident in neonatal sepsis and increased rates of inflammatory disorders [19].

Emerging studies had demonstrated noncoding RNAs, such as lncRNAs and miRNAs, were involved in regulating the progression of human diseases. In neonatal sepsis, multiple miRNAs were reported. For example, microRNA-300/NAMPT regulates inflammatory responses through activation of the AMPK/mTOR signaling pathway in neonatal sepsis [20]. miR-15a/16 are upregulated in the serum of neonatal sepsis patients and inhibit the LPS-induced inflammatory pathway [21]. lncRNAs were a type of ncRNAs longer than 200 bps. Emerging evidences showed lncRNAs played important roles in human diseases, such as diabetes, multiple cancers, and neurodegenerative diseases. A recent study showed lncRNA SNHG16 reverses the effects of miR-15a/16 on the LPS-induced inflammatory pathway in neonatal sepsis [6]. However, the molecular functions of lncRNAs in neonatal sepsis remained unclear. This study identified 28 upregulated lncRNAs and 61 downregulated lncRNAs in neonatal sepsis. Coexpression analysis were used to identify key lncRNAs, including HS.294603, LOC391811, C12ORF47, LOC729021, HS.546375, HNRPA1L-2, LOC158345, and HS.495041. Bioinformatics analysis showed these lncRNAs were involved in regulating ribosome, T cell receptor signaling pathway, RNA degradation, insulin resistance, ribosome biogenesis in eukaryotes, and hematopoietic cell lineage.

In this study, there also existed some limitations. Firstly, more samples were needed considering the small sample size in the present study. Secondly, further experimental validation would be required for future verification. Moreover, specific functions of those dysregulated circRNAs had not been further excavated in this study. Therefore, the further researches with a larger samples group should be performed and more experimental validation and much deeper analysis were still needed in the near future.

## 5. Conclusion

In conclusion, the present study identified a total of 1128 upregulated mRNAs, 1008 downregulated mRNAs, 28 upregulated lncRNAs, and 61 downregulated lncRNAs in neonatal sepsis. Then, we constructed PPI networks to identify key regulators in neonatal sepsis, including ITGAM, ITGAX, TLR4, ITGB2, SRC, ELANE, RPLP0, RPS28, RPL26, and RPL27. lncRNA coexpression analysis showed HS.294603, LOC391811, C12ORF47, LOC729021, HS.546375, HNRPA1L-2, LOC158345, and HS.495041 played important roles in the progression of neonatal sepsis. Bioinformatics analysis showed DEGs were involved in the regulation cellular extravasation, acute inflammatory response, macrophage activation of NF-kappa B signaling pathway, TNF signaling pathway, HIF-1 signaling pathway, Toll-like receptor signaling pathway, and ribosome, RNA transport, and spliceosome. lncRNAs were involved in regulating ribosome, T cell receptor signaling pathway, RNA degradation, insulin resistance, ribosome biogenesis in eukaryotes, and hematopoietic cell lineage. We thought this study provided useful information for identifying novel therapeutic markers for neonatal sepsis.

## Data Availability

All the data were reserved and can be accessed in GSE25504 (https://www.ncbi.nlm.nih.gov/geo/query/acc.cgi?acc=GSE25504).
